# Uncovering the molecular signature of feline diffuse iris melanoma through transcriptomic analysis of disease severity

**DOI:** 10.1038/s41598-025-09632-5

**Published:** 2025-07-19

**Authors:** D. Kayes, B. Blacklock, R. McGeachan, E. Scurrell, K. Donnelly, L. Murphy, A. Fawkes, R. Clark, A. Meynert, H. Becher, R. Pittaway, G. Fricker, R. Tetas Pont, A. Suárez-Bonnet, K. L. Bowlt Blacklock

**Affiliations:** 1 The Hospital for Small Animals, Royal (Dick) School of Veterinary Studies, The University of Edinburgh, Edinburgh, UK; 2 Cytopath Veterinary Pathology, Ledbury, UK; 3 MRC Human Genetics Unit, Institute of Genetics and Cancer, The University of Edinburgh, Edinburgh, UK; 4 Edinburgh Clinical Research Facility, The University of Edinburgh, Edinburgh, UK; 5 Institute of Genetics and Cancer, The University of Edinburgh, Edinburgh, UK; 6 The Roslin Institute, The University of Edinburgh, Edinburgh, UK; 7 Dick White Referrals, Six Mile Bottom, UK; 8 Royal Veterinary College, Queen Mother Hospital for Animals, Hatfield, UK

**Keywords:** Feline diffuse iris melanoma, Melanoma metastasis, Transcriptome, Gene expression, Uveal melanoma, Cancer genetics, Eye cancer, Metastasis, Oncogenes, Tumour biomarkers, Tumour immunology, Oncology, Diseases, Eye diseases, Cancer genetics, Gene expression, Genetic markers

## Abstract

Feline diffuse iris melanoma (FDIM) is the most common primary ocular tumour in cats, with metastatic disease occurring in 19–63% of patients. Greater intraocular invasion correlates with increased mortality. No effective therapeutics exist for metastatic FDIM, partly due to a lack of known molecular targets associated with aggressive tumour behaviour. Here, we define the transcriptomic landscape of FDIM in treatment-naïve cats using bulk RNA sequencing on laser capture microdissection and core biopsy specimens from formalin-fixed paraffin-embedded tissue. Samples included ‘iris melanosis’ (dysplastic melanocytes confined to the anterior iris; n = 7), ‘early FDIM’ (neoplastic melanocytes confined to the iris stroma; n = 13), and ‘late FDIM’ (neoplastic infiltration into the ciliary body and sclera; n = 13). Iris melanosis exhibited genetic overlap with early FDIM, supporting its reclassification as ‘melanoma in situ’. Early FDIM showed upregulation of genes linked to tumour initiation, immune recruitment, and motility (e.g., *STOX1, PEG3, XIAP, MCAM, VIM*). Late FDIM exhibited immune microenvironment remodelling, immune evasion, and apoptosis inhibition (e.g., *BIRC2, BIRC5, CCL2, HAVCR2*), with downregulation of *FOX1, FOXC2*, and *SOX11*. These results provide critical biomarkers of disease severity, which may aid in the development of more accurate prognostic tests and more effective targeted therapies for FDIM.

## Introduction

Feline diffuse iris melanoma (FDIM) is the most common primary ocular tumour in the cat, leading to significant infiltrative destruction of the globe, glaucoma, and death secondary to metastatic disease^1–3^. FDIM arises from melanocytes lining the anterior surface of the iris^4^.

Iris melanosis, defined as dysplastic melanocytes lining the anterior iris in up to three layers, is considered a benign precursor lesion, with neoplastic transformation currently characterised by invasion of the underlying iris stroma^4^. Early FDIM, where there is no evident thickening of the iris, can be clinically indistinguishable from iris melanosis^5^. Iris biopsy has been described as a useful adjunctive diagnostic tool for differentiating between iris melanosis and early FDIM^4^.

The metastatic potential of FDIM is significant, with metastasis occurring in 19–63% of patients. The rate of metastasis is proportional to the severity of ocular invasion^3–7^. Patients with early FDIM, where neoplastic melanocytes are confined to the iris and trabecular meshwork, have similar survival times as age-matched control cats^6^. In contrast, patients with late FDIM, where neoplastic melanocytes infiltrate into the iris, ciliary body and sclera, have decreased survival times and an increased metastatic rate^6,7^. Currently, there are no effective treatment options for metastatic FDIM, making early enucleation the preferred intervention to prevent metastasis^5^. Diode laser ablation has been proposed as an alternative to enucleation, however, data regarding its impact on the risk of metastatic disease is lacking^8^.

The genetic and transcriptomic landscape of FDIM remains largely unexplored. A pilot study using targeted quantitative real-time polymerase chain reaction (RT-qPCR) discovered dysregulation in key genes, including *KIT, LTA4, GNAQ, GNA11, BRAF* and *RASF1* in cats with FDIM^9^. These findings suggest that FDIM may share genetic mechanisms with human uveal melanoma (UM), highlighting the potential for cats to serve as a valuable model for studying human disease. Despite this promising insight, the transcriptomic landscape of FDIM has yet to be comprehensively investigated.

Like FDIM, UM exhibits a high metastatic rate, with up to 50% of patients developing metastatic disease^10–12^. Metastasis in UM is typically catastrophic, with the majority of patients succumbing to the disease within 48 months of diagnosis of metastasis^13^. In contrast to FDIM, UM typically affects the posterior uvea, with iris melanoma occurring infrequently^14^. Human iris melanoma shows a lower metastatic potential, with a reported rate of up to 11%. Among patients with confirmed metastasis, the one-year survival is approximately 15%^15^.

The objective of this study was to characterise the transcriptomic landscape of FDIM in order to identify gene expression pathways linked to disease severity. Using formalin-fixed, paraffin-embedded and laser microdissected tissue from patients with iris melanosis, early FDIM and late FDIM, we show for the first time that iris melanosis is a malignant precursor lesion, with genetic overlap with early FDIM. The transcriptomic changes that are associated with FDIM initiation and evolution to a malignant tumour phenotype are elucidated, identifying potential novel therapeutic and prognostic markers.

## Results

### Late feline diffuse iris melanoma is associated with increased metastasis and decreased survival

**Fig. 1:**
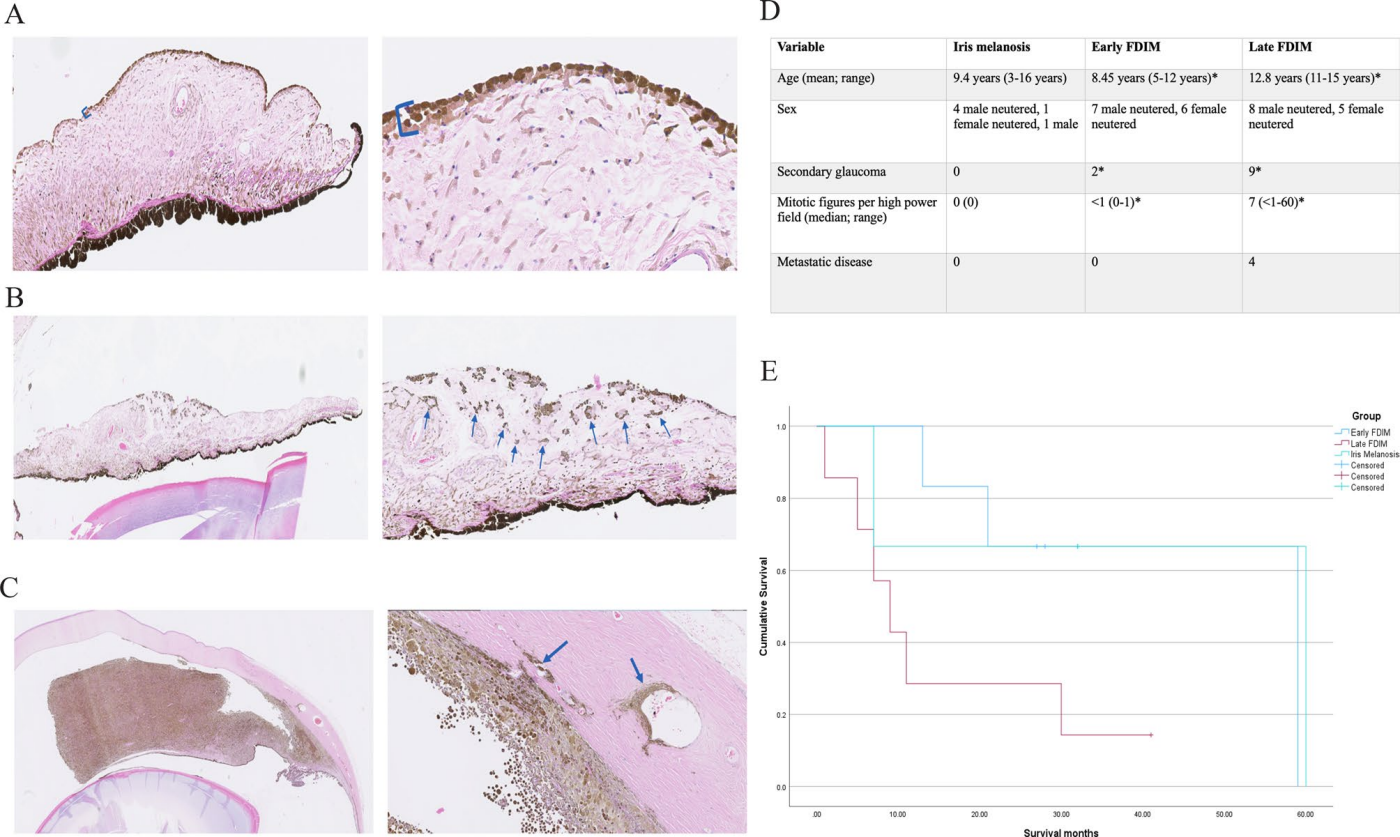
Late feline diffuse iris melanoma (FDIM) is a more locally invasive tumour associated with metastatic disease and reduced survival times. Haematoxylin and eosin staining showing the histological progression from iris melanosis (**A**), to early FDIM (**B**) and then late FDIM (**C**). Iris melanosis (**A**) is characterised by dysplastic melanocytes lining the anterior iris stroma in up to three layers (blue bracket). Progression to early FDIM (**B**) occurs with invasion of the underlying stroma (blue arrows). In late FDIM (**C**), neoplastic melanocytes infiltrate the iris, ciliary body and sclera. The blue arrows highlight invasion of the scleral venous plexus. The overview of the clinicopathologic data from the study (**D**) shows that cats with late FDIM were significantly (*p* < 0.05) older, had higher mitotic activity, and were the only cats with metastatic disease. Statistically significant differences are indicated by * Kaplan–Meier survival analysis (**E**) revealed a significantly (*p* = 0.04) reduced survival time for cats with late FDIM.

We recruited seven cats with iris melanosis (Fig. 1A), 13 cats with early FDIM (Fig. 1B), and 13 cats with late FDIM (Fig. 1C). Clinicopathologic data are summarised in Fig. 1D and Supplementary Table 1. In our dataset, patients with late FDIM were significantly older individuals (*p* < 0.001), had a higher incidence of secondary glaucoma (*p* = 0.005) and tumour histopathology exhibited significantly higher numbers of mitotic figures per high power field (*p* < 0.001) compared to cats with early FDIM.

All patients with late FDIM showed marked local disease extension (Supplementary Table 1), including into the iridocorneal angle or trabecular meshwork (3/13 cats, 23%), the scleral venous plexus (9/13 cats, 69%), choroid (5/13 cats, 38%), or episcleral tissues (3/13 cats, 23%) with additional extension into the extraocular muscles in one cat (1/13, 8%).

Metastatic disease to the liver, spleen or lungs was confirmed or suspected in 4/13 (31%) patients with late FDIM and no patients with early FDIM or melanosis (Fig. 1D and Supplementary Table 1).

Survival data was available for three cats with iris melanosis, six cats with early FDIM and seven cats with late FDIM. Kaplan–Meier survival analysis (Fig. 1E) showed a statistically significant reduction in survival for patients with late FDIM compared to those with early FDIM (*p* = 0.044), with a median survival time of 9 and 27.5 months, for late and early FDIM, respectively.

### Gene expression overlap of melanosis, early and late FDIM, reflect the progressive severity of the disease

**Fig. 2:**
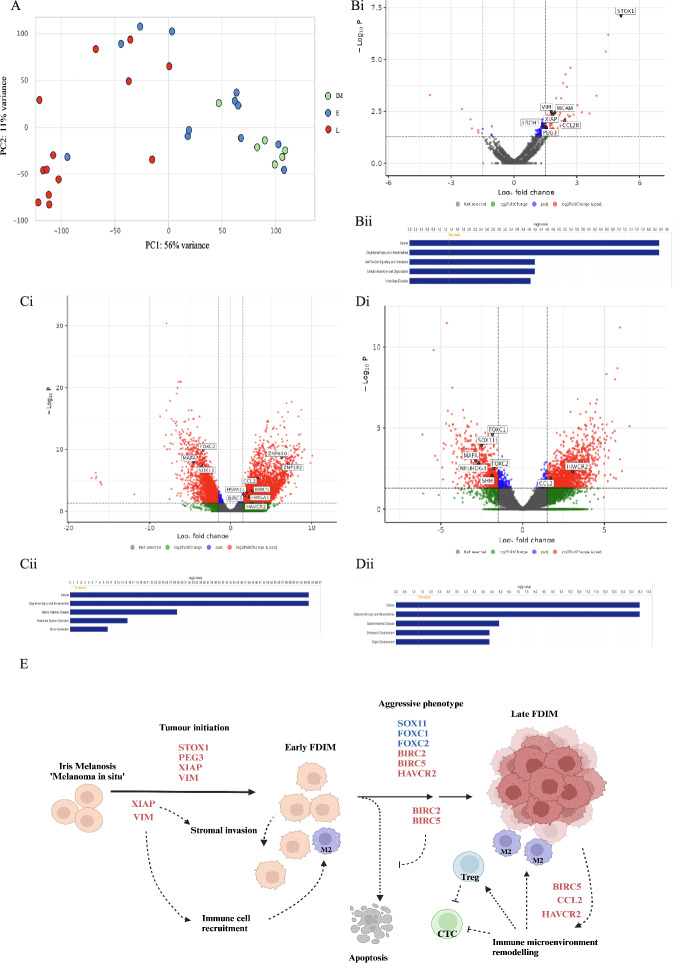
Transcriptomic analysis of feline diffuse iris melanoma (FDIM). The principal component analysis (PCA) plot grouping samples by the top 500 transcripts after variance-stabilising transformation of transcript counts (**A**). There is clear and separate clustering of the iris melanosis and late FDIM samples, with the early FDIM samples overlapping both groups, showing that early FDIM is an intermediate disease stage. Volcano plots highlight the number of significantly dysregulated transcripts between early FDIM and iris melanosis (**B**i), late FDIM and iris melanosis (**C**i) and between late and early FDIM (**D**i). A logfold-change threshold was set at − 1.5 and 1.5 and an adjusted P-value threshold of 0.05 was used. Ingenuity pathway analysis identified cancer and organismal injury and abnormalities to be the top dysregulated disease pathways between early FDIM and iris melanosis (**B**ii), late and iris melanosis (**C**ii) and late and early FDIM (**D**ii). The disease progression of FDIM is associated with key molecular events (**E**). Tumour initiation is associated with upregulation of *STOX1, PEG3, XIAP* and *VIM*, increasing invasive tendencies and immune cell recruitment. Progression to late FDIM is characterised by downregulation of *SOX11, FOXC1* and *FOXC2*, likely leading to a more dedifferentiated and plastic cellular phenotype. Significant upregulation of *BIRC2* and *BIRC5* leads to inhibition of apoptosis. Additionally, upregulation of *BIRC5, CCL2* and *HAVCR2 *lead to immune-microenvironment remodelling, associated with the M2 (immunosuppressive) tumour associated macrophages as well as T-regulator (Treg) cells and inhibition of cytotoxic T-cells (CTC), aiding immune escape. Created in BioRender. Kayes, D. (2025) https://BioRender.com/m39r385.

In this study, we sought to elucidate the molecular mechanisms underpinning disease severity from melanosis to early and late FDIM in cats (Fig. 2). Initially, we examined gene expression clustering, which revealed distinct and separate clustering of late FDIM and iris melanosis samples, reflecting significant differences in their gene expression profiles (Fig. 2A). In contrast, early FDIM samples exhibited overlap with both groups, suggesting a transitional transcriptomic landscape during the progression from melanosis to late FDIM. The results suggest that iris melanosis may be a malignant pre-cursor lesion, showing genetic overlap with early FDIM.

### Early FDIM is characterised by upregulation of cancer-associated and immune-modulatory genes

Next, we explored the transcriptomic differences between melanosis to early FDIM. Comparative gene expression analysis revealed 91 upregulated and 7 downregulated genes in early FDIM compared to melanosis (Fig. 2Bi). Among the upregulated genes were those linked to cancer formation (e.g., *STOX1, PEG3, XIAP*) and immune cell recruitment (e.g., *CCL28, VIM*), suggesting a shift towards increased cellular proliferation and remodelling of the immune microenvironment. Key biological themes identified by ingenuity pathway analysis (IPA) included cancer, organismal injury and abnormality, cell–cell signalling and interaction, cellular assembly and organisation, and hereditary disorder (Fig. 2Bii). Notably, we identified significant upregulation of melanoma associated molecules *MCAM* (melanoma cell adhesion molecule) and *LRCH1* (leucine rich repeats and calponin homology domain containing 1) in early FDIM, highlighting their potential as early indicators of disease progression from melanosis to early FDIM.

Thus, we demonstrate that iris melanosis and early FDIM differ in the expression of multiple cancer-associated genes, with alterations in cell replication pathways and modulation of the immune microenvironment.

### Late FDIM shows further, extensive transcriptomic reprogramming and immune modulation

Next, we explored the transcriptomic landscape of late FDIM in comparison to melanosis (Fig. 2C) and early FDIM (Fig. 2D). Late FDIM showed a marked divergence in its molecular profile, with 101 upregulated and 146 downregulated genes compared to early FDIM, and 595 upregulated and 371 downregulated genes compared to melanosis. These findings demonstrate that the molecular profile of late FDIM diverges significantly from earlier disease stages.

Compared to early FDIM, late FDIM demonstrates advanced disease stages which are characterised by transcriptional reprogramming and immune modulation.

Downregulation of developmental transcription factors, including *FOXC1, FOXC2*, and *SOX11*, suggests increased differentiation and proliferation activity. Significant upregulation and downregulation of multiple genes involved in olfactory receptor pathways and Hedgehog signalling networks were observed, reflecting widespread disruption of cellular communication and developmental signalling pathways. Genes implicated in embryonic and organ development, including *SHH*, *NEUROG3*, and *MAFA*, were downregulated, while immune-related genes like *CCL2* and *HAVCR2* were upregulated, suggesting immune modulation and alterations within the tumour microenvironment. The transition from melanosis to late FDIM represents a profound biological shift, particularly in processes associated with tumorigenesis, cellular differentiation, and metabolic stress. Cancer pathway analysis showed that non-haematological solid tumour pathways and malignant neoplasm formation dominate late FDIM, with dysregulation of key transcription modulators, including members of the *ZNF* family, *HMGA1*, and *SOX* genes. Immune evasion mechanisms were also evident, with alterations in genes like *CCL2* and *HAVCR2*, alongside oncogenes such as *BIRC3* and *BIRC5*, highlighting changes in apoptosis and survival pathways (Fig. 2E). Stress response genes, including *HSPA1L*, reflect heightened cellular stress and metabolic demands.

Consistent activation of cancer-related pathways, such as *EIF2* signalling and translation elongation, further underscores the aggressive nature of late FDIM. Dysregulation of heat shock proteins and ribosomal proteins in late FDIM aligns with increased cellular stress and heightened metabolic activity. Finally, potential biomarkers associated with advanced disease and therapeutic targets were identified, including *MCAM*, *BIRC5*, and *FOXC2*, which may offer new avenues for early detection and intervention in this aggressive disease.

## Materials and methods

Ethical approval was provided by the Veterinary Ethical Review Committee (VERC), The Royal (Dick) School of Veterinary Studies (67.21) and the Clinical Research Ethical Review Board (CRERB), The Royal Veterinary College (URN 2023 2236-2). Informed consent was obtained from the caregivers of all cats included in the study. All experimental methods were carried out in accordance with relevant guidelines, including the ARRIVE guidelines (https://arriveguidelines.org).

### Formalin-fixed, paraffin-embedded (FFPE) biosample collection

Thirty-three archived formalin-fixed, paraffin-embedded (FFPE) feline eyes that were enucleated for reasons unrelated to the study between 1st January 2013 and 1st January 2023 were selected based on morphologic diagnosis of iris melanosis (dysplastic melanocytes confined in up to 3 layers lining the anterior iris stroma; n = 7), ‘early FDIM’ (neoplastic melanocytes confined to the iris stroma; n = 13), and ‘late FDIM’ (infiltration of neoplastic melanocytes through the iris stroma, ciliary body and sclera; n = 13). FFPE blocks were collected from Cytopath Veterinary Pathology, Dick White Referrals, The Royal Veterinary College and Royal (Dick) School of Veterinary Studies. Cats with a history of any prior treatment for any tumour type, or previous history of melanoma were excluded.

### Clinicopathological information

Clinical and phenotypic data collected including patient age (years), sex, breed, age of the FFPE block (months), patient metastatic status at the time of presentation, patient survival after the initial diagnosis (months), development of metastasis and/or recurrence, and any adjuvant therapy provided.

### Laser capture microdissection (LCM)

2.5 μm Haematoxylin and Eosin (H&E) stained sections were prepared from the FFPE blocks and digitally scanned. A diagnosis of melanosis, early FDIM or late FDIM was confirmed by an RCVS specialist in veterinary pathology (ES), and extent of the neoplastic population delineated. In melanosis samples, the dysplastic melanocyte population on the anterior iris surface was delineated. In early FDIM samples, the entire population of neoplastic cells was delineated. In late FDIM samples, neoplastic cells were selected from invasive fronts, specifically neoplastic cell populations that were invading the sclera and/or scleral venous plexus. LCM was performed routinely using a ZEISS™ PALM MicroBeam Laser Microdissection unit (Carl Zeiss Microscopy GmbH, Carl-Zeiss-Promenade 10, 07745 Jena, Germany). The LCM tissue was stored on ice and RNA extraction performed using the Covaris E220 Evolution Focused Ultrasonicator and truXTRAC^®^ FFPE RNA microTUBE Kit—Column (Covaris Ltd, Woddington, Brighton, UK) according to a previously published protocol^16^.

Total RNA was characterised using RNA 6000 Pico kit on the Agilent 2100 Electrophoresis Bioanalyser (Agilent Technologies Inc., 5301 Stevens Creek Blvd, Santa Clara, CA, 95051, USA).

### Library preparation

First-strand cDNA was generated from 50 ng of each total RNA sample using the SMARTer^®^ Stranded Total RNA-Seq Kit v2—Pico Input Mammalian kit (Clontech Laboratories Inc., Mountain View, CA, USA). Due to the high level of expected RNA degradation, no fragmentation was used. Illumina-compatible adapters and indexes were then added via 5 cycles of PCR. The SMARTer kit incorporates SMART^®^ (**S**witching **M**echanism **A**t 5’ end of **R**NA **T**emplate) cDNA synthesis technology and the directionality of the template-switching reaction preserves the strand orientation of the original RNA, making it possible to obtain strand-specific sequencing data from the synthesized cDNA. AMPure XP beads (Beckman Coulter, Brea, CA, USA) were then used to purify the cDNA library. Depletion of ribosomal cDNA (cDNA fragments originating from highly abundant rRNA molecules) was performed using ZapR v2 and R-probes v2 specific to mammalian ribosomal RNA and human mitochondrial rRNA. R-probes bind to library fragments originating from rRNA (18S and 28S) and mitochondrial rRNA (m12S and m16S) and ZapR cleaves these fragments. Uncleaved fragments were then enriched by 16 cycles of PCR for the LCM samples and negative control or 13 cycles for the core samples before a final library purification using AMPure XP beads.

### Library quality control

Libraries were quantified with the Qubit 2.0 Fluorometer and the Qubit dsDNA HS assay kit (Thermo Fisher Scientific, Waltham, MA, USA) and assessed for quality and size distribution of library fragments using the Agilent 2100 Electrophoresis Bioanalyser and the DNA High Sensitivity Kit. The negative control RNA generated a similar quantity of library as the experimental samples. Oneiris melanosis sample was excluded due to poor library mapping rates.

### Sequencing

Sequencing (2 × 100) was performed on the NextSeq 2000 platform (llumina Inc., San Diego, CA, USA) using NextSeq 2000 P3 Reagents (200 Cycles). Libraries were combined in an equimolar pool based on Qubit and Bioanalyser assay results and each pool was sequenced on a P3 flow cell. PhiX Control v3 (Illumina Inc.) was spiked in at a concentration of 1% to allow troubleshooting in the event of any issues with the run.

### Statistical analysis and graphical display of the results

*Alignment and Gene-Level Counts:* RNA-seq data were processed using the nf-core ‘rnaseq’ pipeline v3.8.1^17–26^. In brief, samples were aligned via STAR v2.6.1d, and gene-based counts produced using Salmon v.1.5.2. Feline data were aligned to the Felis_catus_9.0 reference genome, annotated using the corresponding GTF file for build accession GCA_000181335.4.

*Unsupervised Clustering:* Unsupervised consensus clustering of expression data was performed using the R Bioconductor package, ‘cola’^27^. Prior to clustering, gene level count data were subject to a variance stabilising transformation using DESeq2^28^, and the resulting matrix was used as an input. Five different clustering algorithms were tested, evaluating two to six clusters in each case. The cola algorithm resamples count data a fixed number of times, repeating the clustering process on each iteration. The optimum clustering strategy was selected on the stability of the resulting clusters; the method by which samples cluster most consistently.

*Differential Expression Analysis:* Differential expression analysis was performed using DESeq2. Models were fitted treating the clusters identified by cola, sex, and, where appropriate, batch as factors. Log fold change (logFC) estimates were produced using the apeglm shrinkage method^29^, which is intended provide more robust estimates in the event of high within-group variability. Shrunken logFC estimates were accompanied by s-values^30^ an aggregate false sign rate which are broadly analogous to q-values. P-values were also computed and adjusted using the independent hypothesis weighting (IHW) method^31^. Results were annotated using biomaRt^32^, and volcano plots generated using MaGIC Volcano Plot Tool^33^.

*Analysis and graphical display of the results*: Unsupervised hierarchical clustering was performed and differentially expressed genes identified between each of the three cohorts (defined as logFC > 1.5 and adjusted p-value < 0.05). Pathway overrepresentation analyses was based on Kyoto Encyclopedia of Genes and Genomes (KEGG), and Gene Ontology-Biological Process (GO:) databases. Pathway analysis and graphical display of the data was performed using QIAGEN Ingenuity Pathway Analysis (IPA; QIAGEN Inc., https://digitalinsights.qiagen.com/IPA) ^34–37^.

A Mann–Whitney U test (Wicoxon rank sum test) and Fisher’s Exact test was performed to compare clinical data between each cohort using R (version 4.4.2) (R Core Team (2024). _R: A Language and Environment for Statistical Computing_. R Foundation for Statistical Computing, Vienna, Austria. < https://www.R-project.org/ >).

## Discussion

Our findings underscore the aggressive nature of late FDIM, with higher metastatic potential and a reduced survival compared to early FDIM. The reduced survival time should be interpreted with caution, as cats with late FDIM were older, and data were not compared to age-matched controls.

In this study, we sought to identify the gene expression changes that may be responsible for disease initiation and then to evolution of an aggressive late-stage neoplastic phenotype. We found that early FDIM was associated with upregulation of *STOX1, PEG3, XIAP* and *VIM*. Subsequent decreased expression of *SOX11, FOXC1, FOXC2* and increased expression of *BIRC2, BIRC5* and *HAVCR2* was associated with a more severe, late-stage phenotype.

Early FDIM was associated with the upregulation of *XIAP* (X-linked inhibitor of apoptosis protein) and *VIM* (vimentin) suggesting an increased epithelial-mesenchymal transition (EMT) and apoptosis resistance during tumour initiation. EMT is a process in which tumours acquire mesenchymal traits, facilitating tumour cell invasion of the surrounding stroma^38,39^. This hallmark EMT event in early FDIM pathogenesis mirrors that of UM, where *VIM* upregulation correlates with increased tumour invasiveness^40^. *XIAP* plays a dual role in apoptosis resistance and inflammation modulation via the NF-κB signalling pathway while also enhancing melanoma cell migration^38^. *PEG3*, traditionally considered a tumour suppressor when localised to the nucleus, may promote oncogenesis through cytosolic accumulation, inhibiting growth suppressors^41^. Its upregulation in early FDIM may therefore contribute to tumour initiation.

The tumour microenvironment (TME) appears to play a critical role in FDIM severity, mirroring UM. Unlike other solid tumours, increased tumour-associated lymphocytes (TALs) and tumour-associated macrophages (TAMs) in UM contribute to an inflammatory phenotype linked to metastasis and poor survival^42–46^. Monosomy 3 and *BAP1* loss drive M2 macrophage polarisation and proinflammatory cytokine release, particularly *CCL2*, a key driver of monocyte chemotaxis and M2 macrophage differentiation^47^. This promotes an inflammatory TME that inhibits natural killer (NK) cell-mediated cytolysis and induces angiogenesis, facilitating tumorigenesis and metastasis^46^.

*HAVCR2* (encoding the immune checkpoint *TIM-3*) is associated with poor prognosis by suppressing immune responses from macrophages, dendritic cells, NK cells, and Tregs^48,49^. The *GAL9/TIM-3* axis induces cytotoxic T-cell apoptosis and correlates with increased *PD-L1* expression, reducing survival^50^. High *GAL9* levels in aqueous humour further indicate poor prognosis^51^. Additionally, the inflammatory UM phenotype upregulates *PD-1* expression, contributing to resistance to T-cell-mediated tumour destruction^52^*. CCL28* and its receptor CCR10 enhance immune suppression by recruiting Tregs, cancer-associated fibroblasts, and myeloid-derived suppressor cells (MDSCs) ^53^.

We propose that FDIM initiation is associated with immune cell recruitment via *XIAP, CCL28*, and *VIM*. A severe, late-disease state is characterised by upregulation of *CCL2, BIRC5*, and *HAVCR2,* leading to an inflammatory phenotype with inhibition of T-cell-mediated cytolysis, mirroring the malignant behaviour of UM.

Recent advances in immune checkpoint inhibition (ICI), particularly targeting *PD-1* and *CTLA-4*, have significantly improved outcomes in cutaneous melanoma. However, UM exhibits poor responses to ICI monotherapy due to a low tumour mutational burden and an immune-suppressive TME, which limits T-cell activation^54^. The approval of tebentafusp (Kimmtrak), an immune-mobilising monoclonal T-cell receptor against cancer (ImmTAC), has shown promise in UM^55–57^. Tebentafusp functions as a bispecific T-cell engager, directing T cells to lyse tumour cells presenting the melanocyte-specific antigen gp100280-288 via HLA-A02:01. However, this therapy is limited to HLA-A02:01-positive patients^56^.

Given the identified immune landscape modification of FDIM, alternative immune-based therapies should be explored. *CCL2* is prognostic for hepatocellular carcinoma (HCC), where targeting TAMs via *CCL2/CCR2* blockade effectively reduces tumour growth, reverses the immunosuppressive TME, and enhances cytotoxic T-cell responses^58^. Combining *CCR2* antagonism with anti-PD-1 therapy has demonstrated improved tumour responses in solid tumours resistant to ICI monotherapy^59^. This suggests a promising therapeutic avenue for overcoming immune evasion in FDIM.

*TIM-3* has emerged as a novel ICI target, with its inhibition enhancing antigen-specific T-cell responses. *TIM-3* blockade, both as monotherapy and in combination with *PD-1/PD-L1* or *CTLA-4* inhibitors, has shown promise in human solid tumours, including cutaneous melanoma^60,61^. Given *HAVCR2* upregulation in FDIM, *TIM-3* inhibition may improve immune-mediated tumour control in this disease.

Furthermore, the inhibitor of apoptosis *BIRC5* (survivin) is implicated in chemotherapy resistance in UM by reducing apoptosis rates. *BIRC5*-targeted therapy has been proposed to sensitize tumours to treatment and inhibit growth^62^. Vaccination with *BIRC5*-derived peptides has shown promise in metastatic cutaneous melanoma, with survivin-based immunotherapy well tolerated in humans and may have a synergistic effect when combined with existing ICIs^63^.

A surprising finding in our study was the downregulation of *FOXC1, FOXC2* and *SOX11* in late FDIM. *FOXC1* and *FOXC2* are developmental transcription factors critical for embryogenesis and tissue differentiation, especially neural crest and uveal development^64,65^. Specifically, loss-of-function mutations to *FOXC1* in humans are associated with Axenfeld-Rieger Syndrome- a condition characterised by anterior segment dysgenesis of the eye^66^. While *FOXC1* and *FOXC2* are often upregulated in multiple human cancers to drive EMT and metastasis, their downregulation in late FDIM suggests an alternative mechanism in tumorigenesis^64,65,67–69^. One possibility is that *FOXC1/FOXC2* loss results in tumour dedifferentiation, producing a more aggressive and plastic tumour phenotype. A single report has shown that poor *FOXC1* expression was associated with decreased survival in UM, suggesting a similar mechanism of action may exist in UM^70^. The transcription factor *SOX11*, has a context-dependent role in cancer, and appears to be downregulated in late FDIM. In certain human cancers, *SOX11* acts as a tumour suppressor, whereas in others, such as prostate cancer, it promotes proliferation, migration and invasion^71,72^. Research of the role of *FOXC1, FOXC2* and *SOX11* in the context of UM is limited, and further investigation is needed to elucidate the roles of these transcription factors in both FDIM and UM.

In human UM, a PCR-based test (DecisionDx-UM) analyses the expression of 15 genes and accurately stratifies patients into low risk (Class I) and high-risk (Class II) groups, with minimal RNA obtained from fine-needle biopsy specimens^73,74^. Data provided by this study could be used to develop an equivalent assay for FDIM, which could significantly enhance the diagnostic yield of iris biopsy specimens, providing crucial prognostic information and guiding clinical decision-making.

The development of two- and three-dimensional FDIM cell culture lines would facilitate further investigation into the dysregulated pathways identified in this study. Additionally, the generation of FDIM cell cultures would enable validation of potential therapeutic biomarkers and assessment of in vitro susceptibility to existing chemotherapeutic agents.

There were several limitations of the study. The study lacked a control group of normal feline iris melanocytes, for which to compare RNA data. No conclusions can therefore be made as to the gene expression changes from normal melanocytes to iris melanosis. The sample size was small, and future studies should seek to recruit increased numbers of patients. Additionally, age-matched control cats were not included in the analysis of survival data. Finally, the retrospective nature of the study meant that long-term follow-up data was lacking for some cats included in the study.

## Conclusion

In conclusion, our findings offer new insights into the molecular landscape of FDIM, implying that iris melanosis is a malignant precursor lesion with genetic overlap with early FDIM. Based on this, we propose renaming iris melanosis to ‘melanoma in situ’.

This study highlights the molecular complexity of FDIM, revealing potential mechanisms of tumour initiation via immune modulation and EMT, followed by transcriptional dysregulation in late-stage disease characterised by immune evasion and apoptosis resistance. The downregulation of *FOXC1, FOXC2*, and *SOX11* suggests these transcription factors may play a role in maintaining tumour differentiation and regulating metastatic behaviour, warranting further investigation. The role of *CCL2, HAVCR2*, and *BIRC5* in immune modulation and tumour progression present potential therapeutic targets.

Further research is needed to validate the functional roles of these molecular pathways, particularly within the immune microenvironment. Given the similarities identified between FDIM and human uveal melanoma, these findings have broader implications for understanding ocular melanoma pathogenesis across species.

## Supplementary Information

Supplementary Information.

## Data Availability

The datasets generated and analysed during the current study are available in the Sequence Read Archive (SRA,) National Center for Biotechnology Information (NCBI), project reference: PRJNA1238379, available at: https://www.ncbi.nlm.nih.gov/sra/PRJNA1238379.
