# Supplementary material for: Uncovering the molecular signature of feline diffuse iris melanoma through transcriptomic analysis of disease severity
Source: Sci Rep. 2025 Jul 19;15:26218. doi: 10.1038/s41598-025-09632-5 (PMC12276267; doi:10.1038/s41598-025-09632-5)
Supplement: Supplementary file 1 — Supplementary Information. [file 41598_2025_9632_MOESM1_ESM.docx]

**Supplementary Data**

**Supplementary Table 1.** Summary of the clinicopathologic data including patient signalment, histopathologic findings, staging information and follow-up data. Domestic shorthair (DSH), British shorthair (BSH).

| Cat | Cohort | Age (at time of enucleation | Breed | | Sex | Histopathological characteristics | Mitotic figures (per high power field; 2.37mm^2^) | Staging information | Metastatic status at presentation | Deceased^*^ | Survival (months) | Development of metastases or recurrence^*^ |
| --- | --- | --- | --- | --- | --- | --- | --- | --- | --- | --- | --- | --- |
| 1 | Early | 11 years | DSH | Male neutered | | Polygonal to spindeloid cells lining the anterior surface in 1-3 layers and focally infiltrating the anterior iris stroma. | <1 | Not available | Not available | N |  | N |
| 2 | Early | 6 years | DSH | Female neutered | | Polygonal to round cells lining the anterior surface in 1-3 layers and focally infiltrating the anterior iris stroma. | 0 | Thoracic radiographs. | Not evident on radiographs; full staging information not available | N |  | N |
| 3 | Early | 5 years | DSH | Female neutered | | Polygonal melanocytes lining the anterior surface in 1-3 layers and focally infiltrating the anterior iris stroma. | <1 | Thoracic radiographs. | Not evident on radiographs; full staging information not available | N |  | N |
| 4 | Early | 5 years | DSH | Female neutered | | Coarsely pigmented melanocytes lining the anterior surface of the iris in 1-3 layers and focally infiltrating the anterior iris stroma. Secondary glaucoma. | 0 | Thoracic radiographs, abdominal ultrasound scan. | N | N |  | N |
| 5 | Early | 6 years | Ragdoll | Male neutered | | Oval to polygonal cells expanding the entire iris stroma. | 1 | Thoracic radiographs, abdominal ultrasound scan. | N | Not available | Not available | Not available |
| 6 | Early | 5 years | Persian | Male neutered | | Polygonal to elongate melanocytes lining the anterior surface of the iris in 1-3 layers and focally infiltrating the iris stroma. | 0 | Not available | Not available | Not available | Not available | Not available |
| 7 | Early | 11 years | DSH | Male neutered | | Large polygonal round melanocytes lining the anterior surface of the iris in 1-3 layers and focally infiltrating the iris stroma. | 0 | Thoracoabdominal computed tomography. | N | Y | 21 | Euthanised due to unknown bladder mass. |
| 8 | Early | 12 years | DSH | Male neutered | | Polygonal to elongate melanocytes lining the anterior surface of the iris in 1-3 layers and focally infiltrating the iris stroma. | 0 | Thoracic radiographs, abdominal ultrasound scan. | N | Y | 21 | Euthanised due to aortic thromboembolism (presumed unrelated). |
| 9 | Early | 12 years | DSH | Male neutered | | Large polygonal round melanocytes lining the anterior surface of the iris in 1-3 layers and focally infiltrating the iris stroma. | 0 | Not available | Not available | Not available | Not available | Not available |
| 10 | Early | 9 years 8 months | DSH | Female neutered | | Spindeloid cells with moderate anisocytosis and mild anisokaryosis moderately expanding the iris in discrete sheets. Secondary glaucoma. | 0 | Thoracoabdominal computed tomography. | N | Not available | Not available | Not available |
| 11 | Early | 10 years 2 months | DSH | Female neutered | | Round to spindeloid cells extending through 75% of the iris stroma. | 0 | Not available | Not available | Not available | Not available | Not available |
| 12 | Early | 12 years | DSH | Male neutered | | Spindeloid melanocytes lining the anterior surface of the iris in 1-3 layers and focally infiltrating the iris stroma. | <1 | Fine needle sample of the ipsilateral submandibular lymph node consistent with a reactive lymphocytosis. | Not available | Y | 59 | Unknown. Euthanised due to development of ascites (unknown cause). |
| 13 | Early | 5 years | DSH | Female neutered | | Round to polygonal cells with mild anisocytosis and anisokaryosis broadly expanding the iris leaflets multifocally. | 0 | Not available | Not available |  |  | Not available |
| 14 | Late | 15 years | DSH | Male neutered | | Densely packed epithelioid cells invading the iris, ciliary body, iridocorneal drainage angle, peripheral choroid, sclera, peripheral cornea scleral venous plexus, episcleral and bulbar conjunctiva. | 39 | Not available | Not available | Y | 1 | Suspected local recurrence- mass noted on the hard palate and ramus of the mandible ipsilateral to enucleated eye. |
| 15 | Late | 12 years | Selkirk Rex | Male neutered | | Polygonal to spindeloid cells invading the iris, ciliary body, iridocorneal drainage angle, scleral venous plexus and episcleral tissue. Secondary glaucoma. | 13 | Not available | Not available | Y | 9 | Unknown. Patient developed unusual behaviour and reportedly was licking a brick wall 8 months after the enucleation and reportedly died 3 weeks later. |
| 16 | Late | 12 years | Ragdoll | Male neutered | | Polygonal to spindeloid cells invading the iris, ciliary body, iridocorneal drainage angle and scleral venous plexus Secondary glaucoma. | 4 | Not available | Not available | N |  | N |
| 17 | Late | 13 years | DSH | Male neutered | | Spindeloid cells invading the iris, ciliary body, iridocorneal drainage angle, scleral venous plexus, peripheral cornea, filling the anterior chamber and vitreous and extending through the sclera into the extraocular connective tissue, bulbar conjunctiva and extraocular muscles. | 16 | Not available | Not available | Y | 5 | Y |
| 18 | Late | 12 years 8 months | DSH | Male neutered | | Polygonal cells invading the iris, ciliary body, iridocorneal angle, choroid, scleral venous plexus. Secondary glaucoma. | 20 | Not available | Not available | Y | 3 | Suspected- abdominal ultrasound scan identifying hyperechoic hepatic nodules and thoracic radiographs with military pattern. |
| 19 | Late | 15 years | Exotic shorthair | Male neutered | | Polygonal to spindeloid cells invading the iris, ciliary body, trabecular meshwork, sclera and scleral venous plexus. Marked cellular pleomorhphism, with karyomegaly and intranuclear cytoplasmic inclusions present. Secondary glaucoma. | 29 | Right-lateral thoracic radiograph. | Suspected liver metastasis with ‘large liver mass’ identified radiographically. | Y | 7 | Suspected- liver mass identified at the time of enucleation on radiographs and confirmed via abdominal ultrasound immediately prior to euthanasia. |
| 20 | Late | 12 years | DSH | Female neutered | | Round to oval to spindle-shaped cells invading the iris, ciliary body, iridocorneal angle and sclera. Marked anisocytosis and anisokaryosis present. Secondary glaucoma. | 7 | Thoracic radiographs, abdominal ultrasound. | N | Y | 30 | Confirmed metastasis to the liver and spleen. Thoracic radiographs identified a consolidated left cranial lung lobe and pleural effusion. Fine needle aspirate samples of the liver and spleen consistent with metastatic melanoma. |
| 21 | Late | 14 years | BSH | Female neutered | | Large polygonal to intermediate sized spindeloid cells infiltrating the iris, ciliary body, limbus, iridocorneal drainage angle and sclera. Secondary glaucoma. | <1 | Not available | Not available | Not available | Not available | Not available |
| 22 | Late | 11 years | DSH | Male neutered | | Large polygonal cells with moderate anisocytosis and anisokaryosis infiltrating the iris, ciliary body, sclera and scleral venous plexus. | 2 | Not available | Not available | Not available | Not available | Not available |
| 23 | Late | 14 years | DSH | Female neutered | | Large polygonal to intermediate sized spendeloid cells invading the iris, ciliary body, sclera and scleral venous plexus. Mild anisokaryosis. | <1 | Not available | Not available | Not available | Not available | Not available |
| 24 | Late | 11 years 1 month | DSH | Female neutered | | Round to polygonal cells invading the iris, ciliary body, anterior choroid, trabecular meshwork and sclera. Moderate anisocytosis and anisokaryosis. Secondary glaucoma. | 60 | Thoracoabdominal computed tomography. | Nodular liver mass. Fine needle aspirate samples consistent with metastatic melanoma. | Y | Not available | Confirmed hepatic metastasis at time of enucleation. |
| 25 | Late | 12 years | DSH | Female neutered | | Oval, round or epitheioid cells invading the iris, ciliary body and trabecular meshwork. A large cluster of neoplastic cells were identified within the choroid (intravascular neoplastic embolus). Marked anisocytosis and moderate anisokaryosis. Secondary glaucoma. | 3 | N | Not available | N | Not available | Not available |
| 26 | Late | 12 years 10 months | DSH | Male neutered | | Spindeoloid to round cells with mild anisocytosis and anisokaryosis infiltrating the iris, ciliary body and anterior choroid with pigmented cells present in the scleral venous plexus. Secondary glaucoma. | <1 | Not available | Not available | Not available | Not available | Not available |
| 27 | Iris melanosis | 9 years | DSH | Male neutered | | 1-2 layers of melanocytes with coarse granular pigment lining the anterior iris stroma. | 0 | Thoracoabdominal computed tomography. | N | Y | 7 | N |
| 28 | Iris melanosis | 14 years | DSH | Male | | 1-2 layers of melanocytes with coarse granular pigment lining the anterior iris stroma. Mild cellular pleomorphism and anisokaryosis. | 0 | N | Not available | N |  | N |
| 29 | Iris melanosis | 16 years | DSH | Male neutered | | 2-3 layers of melanocytes with open nuclei and small distinct nucleoli lining the anterior iris stroma. | 0 | Thoracic radiographs, abdominal ultrasound. | N | Y | 62 | Not available |
| 30 | Iris melanosis | 3 years | BSH | Female neutered | | 1-2 layers of melanocytes with coarse granular pigment lining the anterior iris stroma. Minimal anisocytosis and anisokaryosis. | 0 | Thoracic radiographs, abdominal ultrasound. | N | Loss of follow-up following enucleation. | Not available | Not available |
| 31 | Iris melanosis | 10 years 5 months | DSH | Male neutered | | 1-2 layers of melanocytes with coarse granular pigment lining the anterior iris stroma. Mild pleomorphism. | 0 | N | Not available | Loss of follow-up following enucleation. | Not available | Not available |
| 32 | Iris melanosis | 4 years | DSH | Male neutered | | 1-2 layers of melanocytes with coarse granular pigment lining the anterior iris stroma. | 0 | N | Not available | Loss of follow-up following enucleation. | Not available | Not available |
|  |  |  |  |  | |  |  |  |  |  |  |  |

*as of August 2023
